# Influence of endometrial thickness on treatment outcomes following in vitro fertilization/intracytoplasmic sperm injection

**DOI:** 10.1186/s12958-016-0222-5

**Published:** 2017-01-05

**Authors:** Ning-Zhao Ma, Lei Chen, Wei Dai, Zhi-Qin Bu, Lin-Li Hu, Ying-Pu Sun

**Affiliations:** Department of Reproductive Medical Center, First Affiliated Hospital of Zhengzhou University, JianShe Dong Road, Erqi District, Zhengzhou, Henan Province People’s Republic of China

**Keywords:** Endometrium, IVF/ICSI, Endometrial thickness, Live birth rate

## Abstract

**Background:**

The study was designed to investigate the roles of endometrial thickness (EMT) at the day of human chorionic gonadotropin (hCG) administration on pregnancy outcomes in a large patient population.

**Methods:**

This retrospective cohort study included 9,952 patients undergoing their first IVF/ICSI with autologous oocytes from January 2011 to January 2015. Patients were divided into three groups based on the EMT (group A:≤8 mm; group B: 9–14 mm and group C:≥15 mm). Live birth rate (LBR), clinical pregnancy rate (CPR), early miscarriage rate (EMR), and ectopic pregnancy rate (EPR) were analyzed. Additionally, the live birth rate was analyzed for patients with single or double gestational sacs.

**Results:**

Significant differences (*p* < 0.05) were detected in the LBRs (30.38%, 45.73% and 54.55% for groups A, B, and C, respectively), CPRs (38.57%, 55.04% and 64.32%, respectively), and EPRs (5.58%, 3.48% and 2.19%, respectively), with thicker endometrial thickness favoring all three parameters. However, no differences were found in the EMRs among the three groups (15.64%, 13.44% and 13.05%, respectively, *p* > 0.05). After adjusting for female age, body mass index (BMI) and endometrial pattern, the multivariate logistic regression analysis demonstrated that the associations between EMT and LBR (adjusted OR: 2.645; 95% CI *2.020–3.464*; *p* < 0.01), CPR (adjusted OR 2.693 95% CI 2.012–3.605 *p* < 0.01), and EPR (adjusted OR: 0.298 95% CI *0.101–0.713*; *p* < 0.05) were significant. Additionally, live birth rates in the double gestational sac group were different (*p* < 0.05) among patients with different EMT (72.73%, 87.28%, and 87.36%, respectively), whereas no difference was found in the single gestational sac group. In the double gestational sac group, LBR was positively correlated with increasing endometrial thickness only in patients with twin pregnancies but not in patients with singletons.

**Conclusions:**

Our study shows that endometrial thickness at the day of hCG administration has an effect on LBR, CPR and EPR, with all three parameters increasing with the EMT. Furthermore, successful twin pregnancies are associated with a thicker endometrium.

## Background

Endometrial receptivity and embryo quality are the two primary factors that influence the in vitro fertilization/intracytoplasmic sperm injection-embryo transfer (IVF/ICSI-ET) pregnancy outcome. As the site of embryo implantation, the endometrium provides optimal environments for early embryo development and implantation following regulation by ovarian estrogen and progesterone. Endometrial receptivity has received increasing attention. Due to the safety and simplicity of ultrasonic examination, an evaluation of EMT provides an important indicator to predict pregnancy outcomes [1–3]. However, the exact influence of EMT at the day of hCG administration on pregnancy outcomes remains controversial due to a lack of large scale systemic studies. Some studies have suggested that the pregnancy rate increases with the increasing of the EMT [4–7], whereas other studies have indicated that the EMT is not correlated with the pregnancy rate [8–11]. One study found the pregnancy rate decreased in patients with a thickened endometrium [12]. Because most of these studies included small sample sizes and did not consider the influence of other factors such as the maternal age, variability of ovulation stimulation protocols, and number of transferred embryos, we designed a retrospective analysis using a large cohort of patients with a defined age undergoing a standard ovulation stimulation protocol and receiving the same number of transferred embryos. Then, we assessed the influence of EMT measured on the day the patients received hCG on the pregnancy outcomes. Additionally, we evaluated the relationship between the EMT and the pregnancy outcomes in patients with single or double gestational sacs.

## Materials and Methods

### Study objective

A retrospective analysis was performed using data from 9,528 infertility patients who underwent IVF or ICSI at our center from January 2009 to January 2015. The inclusion criteria were as follows: 1. patients who underwent their first IVF/ICSI cycle under the age of 38 years; 2. patients who underwent gonadotropin-releasing hormone agonist treatment using the long protocol; and 3. patients who underwent transfer of two high quality embryos. The exclusion criteria were as follows: patients with uterine malformations, intrauterine adhesions, adenomyosis or endometriosis and patients who underwent pre-implantation genetic diagnosis (PGD). This retrospective cohort study was approved by the Ethics Committee of the First Affiliated Hospital of Zhengzhou University.

### Methods

Ovulation stimulation protocol: Based on the controlled ovulation stimulation long protocol [13], gonadotropin-releasing hormone agonist (GnRH-a, triptorelin, Hui Ling, Switzerland, or Diphereline, Ipsen, France) was injected to decrease the serum gonadotropin levels. The serum FSH was suppressed to <5 mIU/mL, LH to <5 mIU/mL, and estradiol to <50 pg/mL, and an evaluation was performed to validate an endometrial thickness <5 mm and a diameter of the largest bilateral ovarian follicle <10 mm. Then recombinant follicle-stimulating hormone (r-FSH, Gonal-F, Merck, Serono, Switzerland; Pouliquen, Merck Sharp & Dohme, USA; Vermont, IBSA, Switzerland) or human menopausal gonadotropin (HMG, Livzon, China, or Menopur, Hui Ling, Switzerland) was injected to stimulate ovulation stimulation. The gonadotropin dosages were adjusted in individual patients based on the follicle size and endocrine level. Ovulation was triggered with a single dose of hCG when the largest follicle exceeded 20 mm in size and the number of follicles greater than 16 mm in size accounted for more than 2/3 of the total follicles. Two embryos were transferred within 3 days after oocyte retrieva. Within 14 days and 18 days of implantation, serum β-hCG levels of >5 IU/mL) were used as a criterion for a successful biochemical pregnancy. On the 35th day after implantation, ultrasonic examination was performed to check for the presence of gestational sacs, embryonic buds and embryonic heartbeats as parameters for a successful clinical pregnancy. A live birth was defined as the delivery of a viable offspring. We separated cleavage stage embryos into four grades in our center [14]. Grades I–II were regarded as high-quality embryos.

Endometrial thickness and pattern determination: a transvaginal ultrasound scan was adopted to evaluate the EMT at the day of hCG injection. The maximum distance between the two outer edges of the endometrial image on a longitudinal section of the uterus observed using a vaginal B-ultrasound was used to determine the endometrial thickness.

Hormone determination: When the diameter of the largest follicle was more than 14 mm, fasting blood was collected daily to determine the LH, estradiol and progesterone levels. All intra-assay and inter-assay variations were less than 10%.

### Dataset

The patients were divided into three groups according to the EMT as follows: thin endometrium (≤8 mm); intermediate endometrium (9–14 mm); and thick endometrium (≥15 mm). On the 35th day after implantation, we recorded the number of gestational sacs. We also analyzed the relationship between the endometrial thickness and LBRs from the 35th day to the delivery of a viable offspring according to the number of gestational sacs. From the 35th day to the day of delivery, the groups were divided into the double and single gestational sac groups.

### Outcome measures

The LBR was the primary outcome. The LBR was defined as the total number of pregnancies that progressed to the delivery of a viable offspring/the total number of transferred cycles. We also calculated the CPR (total number of pregnancies/number of transferred cycles), EMR (cycles of a pregnancy loss before 28 weeks of gestation and all biochemical pregnancies/total number of clinical pregnancy cycles), and the EPR (cycles of the sac outside the uterine cavity/the total number of clinical pregnancy cycles). After the 35th day, the LBR was defined as the number cycles delivering a viable offspring/the number of cycles in the double gestational sac or the single gestational sac groups.

### Statistical analysis

The SPSS17.0 statistical software was used. Patients’ basic parameters, including age, BMI, duration of infertility and infertility diagnosis, are presented for each group. Each continuous variable (e.g., age) was presented as the mean ± SD, whereas categorical variables (e.g., tuber factor) were presented as the n and %.

Categorical variables were compared using the Chi-square test and Fisher’s exact test. Continuous variables were compared using one-way analysis of variance (ANOVA). Multilevel logistic regression was used to assess the relationship between the EMT and the pregnancy outcomes (CPR, LBR and EPR), after adjusting for maternal age, BMI and endometrial pattern. For the categorical covariates, one category served as the reference category, and all other categories were compared to the reference category. In our study, we used the ≤8 mm group as the reference group. The results were expressed as the odds ratio (OR) with 95% confidence intervals (CI) and were tested using the Wald *x* ^*2*^ tests. *p* < 0.05 was considered statistically significant.

## Results

### Patient features

A total of 9,528 patients were involved in this study, including 7,428 IVF and 2,177 ICSI patients. The mean patient age was 30.88 years (SD = 4.92). The mean maternal BMI was 22.37 kg/m^2^ (SD = 3.2). A total of 8,283 patients used recombinant FSH and 1,245 patients used urinary FSH. The study included 8,209 patients with a triple line pattern and 1,319 patients with a non-triple line pattern. Details of the basic characteristics across endometrial thickness brackets are presented in Table 1. The EMT of patients included in the study ranged from 4 to 26 mm.

**Table 1:** Basic characteristics of the 9,528 patients who underwent an initial IVF/ICSI cycle

	Group A (≤8 mm)	Group B (9–14 mm)	Group C (≥15 mm)	*p*
NO.	464	7,539	1,525	
Age (year)	30.44 ± 4.065	29.95 ± 4.367	30.07 ± 4.322	0.060
BMI (kg/m^2^)	21.3 ± 2.32	22.39 ± 3.07	22.33 ± 3.55	0.142
Duration of infertility (year)	3.64 ± 2.5	4.5 ± 3.62	4.6 ± 3.39	0.351
Basal FSH (IU/L)	7.76 ± 2.4	7.63 ± 2.71	7.79 ± 2.5	0.045
Gn (days)	12.09 ± 1.68	12.04 ± 1.64	12.10 ± 1.60	0.342
Gn dosage (IU)	2,163.21 ± 1,263.01	2,149.34 ± 876.44	2,156.46 ± 844.392	0.943
Type of Gn				
Recombinant FSH	83.78%(389)	86.99%(6,558)	87.60%(1,336)	0.101
Urinary FSH	16.22%(75)	13.01%(981)	12.40%(189)	0.101
Endometrial pattern				
Triple line pattern	71.39%(331)	87.54%(6,599)	83.91%(1,279)	0.000
No-triple line pattern	28.61%(133)	12.46%(940)	17.09%(246)	0.000
IVF	81.03%(376)	77.99%(5,880)	76.19%(1,162)	0.096
ICSI	18.96%(88)	22.01%(1,659)	23.81 (363)	0.096
Long agonist protocol	100%(464)	100%(7,539)	100%(1,525)	NS
No. of transferred embryos	2	2	2	NS
Infertility diagnosis				
Tubal factor	57.11%(265)	51.29%(3867)	42.29%(645)	0.000
Male factor	28.23%(131)	38.49%(2902)	40.06%(611)	0.000
Endometriosis, pelvic and uterine factors	5.38%(25)	5.50%(415)	5.83%(89)	0.865
Unexplained and others	9.26%(43)	4.70%(355)	11.80%(180)	0.000

Values are the mean ± SD unless otherwise noted

*BMI* body mass index, *FSH* Follicle-stimulating hormone, *Gn* gonadotropin treatment, *IVF* in vitro fertilization, *ICSI* intracytoplasmic sperm injection, *NS* no significance

Among the three groups with varying endometrial thicknesses, no significant differences were found in the patient age, BMI, duration of infertility, gonadotropin treatment time, dosage of gonadotropin administered, type of gonadotropin, or quantity of transferred embryos. However, differences were observed in the basal FSH and endometrial pattern (Table 1).

### Relationship between EMT and pregnancy outcome

The CPR increased with increasing endometrial thickness, (groups A,:38.57%, B: 55.04%, and C: 64.32%; *p* < 0.001); the LBR also increased (A: 30.38%, B: 45.73%, and C: 54.55% *p* < 0.001). In contrast, the incidence of ectopic pregnancy decreased with increasing endometrial thickness (A: 5.58%, B: 3.48%, and C: 2.19%, *p* < 0.05). No significant difference was observed in the abortion rates in the three groups (A: 15.64%, B: 13.44%, and C: 13.05%; *p* > 0.05) (Table 2).

**Table 2:** Clinical outcomes among three different endometrial thickness groups

Endometrial thickness (mm)	Group A (≤8)	Group B (9–14)	Group C (≥15)	*p*-value
Live birth rate	30.38% (141/464)	45.73%(3416/7539)	54.55%(832/1525)	<0.001
Clinical pregnancy rate	38.57% (179/464)	55.04%(4150/7539)	64.32%(981/1525)	<0.001
Abortion rate	15.64% (28/179)	13.4%(558/4150)	13.06%(131/981)	0.645
Ectopic pregnancy rate	5.58%(10/179)	3.48%(144/4150)	2.19%(21/981)	0.024

Among the 9,528 cases, the thinnest endometrial thickness was 4 mm and led to a heterotopic pregnancy, whereas the thickest endometrial thickness was 26 mm and led to an intrauterine pregnancy.

### CPR, LBR and EPR assessed using logistic regression analysis

Table 3 shows the multilevel logistic regression for the CPR, LBR and EPR results according to the EMT. Adjusting for maternal age, BMI and endometrial pattern, we found that all groups had significantly higher odds of pregnancy than the thinnest group (OR 1.876 95% CI:1.434–2.453, *p* < 0.01; OR 2.693 95%CI: 2.012–3.605, *p* < 0.01). We hypothesized that the odds of a live birth were also higher than those of the reference group (OR 1.701 95% CI: 1.333–2.170*, p* < 0.01; OR 2.645 95% CI: 2.020–3.464, *p* < 0.01). The patients in the ≥15 mm group had a significantly lower risk for ectopic pregnancy than the patients in the reference group (OR 0.298 95% CI: 0.101–0.879, *p* < 0.05) (Table 3).

**Table 3:** Odds ratios for live birth, pregnancy and ectopic pregnancy occurrence rates compared with the reference group

	Live birth rate			Pregnancy rate			Ectopic pregnancy rate		
	P	aOR	95%CI	P	aOR	95%CI	P	aOR	95%CI
A		1 (reference)			1 (reference)			1 (reference)	
B	<0.01	1.701	1.333–2.170	<0.01	1.876	1.434–2.453	0.332	0.632	0.250–1.598
C	<0.01	2.645	2.020–3.464	<0.01	2.693	2.012–3.605	0.028	0.298	0.101–0.879

Adjusted for maternal age, BMI and Endometrial pattern. *P* values were calculated from Wald *x* ^*2*^ tests

### The relationship between EMT and live birth from the 35th day to the delivery of a viable offspring

We further subdivided the patients based on the detection of single or double gestational sacs. For the double gestational sac group, the live birth rate was highest in the patients with a thicker endometrium (72.73%, 87.28%, and 87.36% in groups A, B, and C, respectively; *p* = 0.018). Conversely, no difference was found in the single gestational sac group. We also analyzed the subgroup of patients with a double gestational sac and found that the LBR increased with an increase in the EMT in patients with two sacs and who gave birth to twins (2 → 2 group) (A: 50%, B: 69.79%, and C: 71.42% *p* < 0.05). However, the patients with two sacs but a singleton birth exhibited no significant differences in LBR among three groups with different EMTs (A: 22.72%, B: 17%, and C:13.70%; *p* > 0.05) (Table 4).

**Table 4:** The effect of endometrial thickness on live birth rate in patients with single or double gestational sacs

		Group A (≤8)	Group B (9–14)	Group C (≥15)	*P*-value
Double gestational sac		72.73%(32/44)	87.28%(1263/1447)	87.36%(318/364)	0.018
	2 → 2	50.00%(22/44)	69.79%(1010/1447)	71.42%(260/364)	0.014
	2 → 1	22.72%(10/44)	17.00%(247/1447)	13.70%(50/364)	0.246
Single gestational sac		75.00%(102/136)	78.50%(2148/2736)	82.09%(509/620)	0.071

2 to 2: patients with double gestational sacs and twin birth; 2 to 1 patients with double gestational sacs and single birth

In the current study, there were eight patients had three gestational sacs. In our center, patients with a triplet pregnancy usually undergo multi-fetal pregnancy reduction (MFPR). We also recommend patients with a twin pregnancy to receive fetal reduction. In this study, we excluded the patients received fetal reduction from group (2 → 1).

## Discussion

We showed that a thin endometrium could have an adverse effect on the CPR, LBR and EPR, but no association was found with the EMR. Furthermore, a thick endometrium was associated with an ability to give birth to twins after the 35th day.

The relationship between the EMT and the pregnancy outcome has long been controversial. Weissman et al. [12] that the pregnancy rate was significantly decreased when the EMT was greater than 14 mm (>14 mm) and that the abortion rate increased concomitant with the EMT. Conversely, a prospective study by Rashidi et al. [8] with 150 subjects showed no difference in the EMT between pregnant females and non-pregnant females; however, the authors found that patients with an endometrium thicker than 12 mm (>12 mm) or thinner than 9 mm (<9 mm) did not become pregnant. Zhao et al. [15] and others hypothesized that a thickening endometrium and the triple line pattern were beneficial to pregnancy but could not predict the pregnancy outcomes. Dietterich et al. [16] also reported that they did not find a relationship between the implantation, pregnancy rate, and EMT. However, Most previous studies were based on retrospective analyses with a small sample population and did not control for other factors that could affect the pregnancy rate. Here, we not only collected a large number of samples from research subjects but also controlled for various confounding factors. To limit the effect of maternal age and stimulation cycles, only the first cycle and patients aged ≤ 38 y were included. Additionally, two high quality embryos were transferred to all patients to control for embryonic factors for a successful pregnancy. Unified down-regulation and ovulation stimulation protocols were also used to reduce the potential effects of ovarian stimulation and ovarian steroids on pregnancy. Additionally, maternal age, endometrial pattern and BMI were treated as potential confounders to control for bias when we studied the influence of endometrial thickness on pregnancy treatment outcomes.

Our study suggested that the CPR and LBR increased concomitant with the increase in the EMT. Additionally, the risk of ectopic pregnancy was decreased. However, no significant difference in the abortion rate was found among the three groups. This result was differed from the result of Weissman et al. [12]. In their studies, age differences were not controlled. The patients in the group with a thickened endometrium might be older than the patients in the group with a thinner endometrium. In a recent meta-analysis on the relationship between EMT and pregnancy rate, Kasius et al. [17] proposed that the reason the conclusions were inconsistent was that we did not consider the influence of patient’s age and the number of oocytes retrieved on the pregnancy rate. Yuan et al. [18] reported that EMT was one independent variable predictive of clinical pregnancy, live birth, spontaneous abortion, and ectopic pregnancy. Although their study also included a large sample, they did not control for confounding factors, including the ovulation stimulation protocols and the transfer of different numbers of embryos. After adjusting for confounders, we also detected an effect of the endometrium on the CPR, LBR and EPR in the multivariate logistic regression analysis.

Kumbak et al. [19] indicated that patients with an endometrium <7 mm had a poor outcome, but the pregnancy rate could be improved by increasing the number of transferred embryos. In our study, the group A also have the lowest CPR, although the reason was unclear. Casper [20] proposed that this finding was associated with the endometrial oxygen concentration. When the endometrium is thin, embryo can implant near the spiral arteries at the basal layer of the endometrium, where the high oxygen concentrations near the basal layer can negatively affect embryo development. With the decline in EMT, the endometrial receptivity inside the uterus decreases, and the embryos can implant outside of the uterine cavity, leading to high incidence of ectopic pregnancy. A retrospective cohort study of 8120 patients by Rombauts, L et al. [21] found that the risk of EPR was 4-fold in patients with an EMT of <9 mm compared with patients with an EMT of >12 mm. However, further studies should examine the relationship between a thin endometrium and endometrial receptivity. Sundstrom [22] reported a case of successful pregnancy in a patient with a 4 mm-thick endometrium on the OPU day. Zhao et al. [2] also described a successful pregnancy in a patient with a 4.8 mm-thick endometrium. Among our study subjects, the thinnest endometrium was 4 mm, which was present in a heterotopic pregnancy; however, the intrauterine pregnancy proceeded after laparoscopic surgery. Therefore, we could not justify denying a transfer due to a thin endometrium. Additionally, our results suggested that a thickened endometrium had no apparent adverse effect on the clinical pregnancy. The pregnancy rate improved when the endometrial thickness increased, which was consistent with the study results of Chen et al. [1]. A recent study showed that a higher CPR was reached when the EMT was >8 mm, and no adverse effect on the clinical outcome was observed when EMT was > 14 mm [23]. Among our study subjects, the thickest endometrium was 26 mm, and the pregnancy was intrauterine.

Different opinions exist concerning the relationship between the endometrial pattern and the pregnancy rate. Chen et al. [1], Rashidi et al. [8], and Merce et al. [24] postulated that no difference existed between the endometrial pattern and the pregnancy rate. Zhao et al. [2] proposed that a triple line endometrial pattern indicated a good pregnancy outcome only when the endometrial thickness was 7–14 mm. The research by Kuc et al. [25] showed that endometrial pattern had a significant effect on pregnancy rate only in patients undergoing the long agonist treatment protocol, and that patients with the triple line pattern had better pregnancy outcomes. To control for the effect of the endometrial pattern, we regarded this variable as a covariant in our study.

Although this study is a retrospective analysis, the large sample size represents a major strength. Certainly, there are several limitations in our study. For instance, selection bias occurred because all samples came from our medical service hospital. Additionally, the measurement of endometrial thickness by different physicians may have introduced differences.

Our study suggested that EMT in the double gestational sac group but not the single gestational sac group had an effect on live births. According to the subgroup analysis of subjects in the double gestational sac group, only the (2 → 2) group was sensitive to the EMT. We concluded that, for the live birth rate after the 35th day, the EMT only had an effect on the double gestational sacs and the birth of twins. The EMT was more important when giving birth to twins. We speculated that twins might need a thicker endometrium.

## Conclusions

In conclusion, our study demonstrated that the EMT was an important factor affecting the outcome of pregnancy. Moreover, the live birth rate tended to rise with an increase in endometrial thickness. Meanwhile, Patients who gave birth to twins with a thick endometrium had higher live birth rates than those with a thin endometrium.

## Abbreviations

BMI: Body mass index
CI: Confidence interval
COS: Controlled ovarian stimulation
CPR: Clinical pregnancy rate
EMR: Early miscarriage rate
EPR: Ectopic pregnancy rate
FSH: Follicle-stimulating hormone
GnRH: Gonadotropinreleasing hormone
hCG: Human chorionic gonadotropin
ICSI: Intracytoplasmic sperm injection
IVF: In vitrofertilization
LBR: Live birth rate
OR: Odds ratio
SD: Standard deviation
